# Population Dynamics of American Bullfrog (*Lithobates catesbeianus*) and Implications for Control

**DOI:** 10.3390/ani12202827

**Published:** 2022-10-18

**Authors:** Byungwoo Chang, Inyoo Kim, Kwanghun Choi, Wonhee Cho, Dongwook W. Ko

**Affiliations:** 1 Department of Forest Resources, Kookmin University, 77 Jeongneung-ro, Seongbuk-gu, Seoul 02707, Korea; 2 Department of Forest, Environment, and Systems, Kookmin University, 77 Jeongneung-ro, Seongbuk-gu, Seoul 02707, Korea; 3 Industry Academic Cooperation Foundation, Kookmin University, 77 Jeongneung-ro, Seongbuk-gu, Seoul 02707, Korea

**Keywords:** invasive species, stage-based population model, STELLA, UAV, wildlife management

## Abstract

**Simple Summary:**

*Lithobates catesbeianus* (American bullfrog) was introduced to South Korea and caused various damage in the Korean natural environment for the past 25 years. Although several management strategies were implemented, the effectiveness of past control decisions is largely unknown. We built a population dynamics model for *L. catesbeianus* in the Onseok reservoir, South Korea, in order to assist managerial decisions. Control scenarios with varying intensities were simulated to evaluate their effectiveness. The population of the American bullfrog in the reservoir was reduced to a manageable level under intensive control of the tadpole stage, using three sets of double fyke nets and 80% direct removal of juvenile and adult stages. According to our results, integrated, intensive, and continuous control is essential for managing the invasive American bullfrog population.

**Abstract:**

*Lithobates catesbeianus* (American bullfrog), known to be one of the notorious invasive species, was introduced to South Korea and has proliferated in the Korean natural environment for the past 25 years. The ecological impact caused by the species is well known, and several management decisions have been implemented to cull its population. However, the effectiveness of past control decisions is largely unknown. We built a population dynamics model for *L. catesbeianus* in the Onseok reservoir, South Korea, using STELLA architect software. The population model was based on the demographics and ecological process of the species developing through several life stages, with respective parameters for survivorship and carrying capacity. Control scenarios with varying intensities were simulated to evaluate their effectiveness. The limitations of isolated control methods and the importance of integrated management are shown in our results. The population of the American bullfrog in the reservoir was reduced to a manageable level under intensive control of the tadpole stage, using three sets of double fyke nets and 80% direct removal of juvenile and adult stages. According to our results, integrated, intensive, and continuous control is essential for managing the invasive American bullfrog population. Finally, our modeling approach can assist in determining the control intensity to improve the efficiency of measures against *L. catesbeianus*.

## 1. Introduction

Invasive species cause significant environmental damage, such as biodiversity loss and the deterioration of ecosystems, leading to negative economic impacts [1]. Once invasive species are introduced, they possibly have competitive advantages over limited resources with native species because of a lack of natural enemies or human control in the new environment [2,3]. Invasive species can be intentionally introduced for various purposes, such as pets, food, recreation, and pest control, or unintentionally through trade shipment [4]. However, most are introduced without prior knowledge of the potential negative impacts on the native ecosystem when released into the natural environment.

The American bullfrog (*Lithobates catesbeianus*) is a notorious invasive species listed among the top 100 of the world’s worst invasive alien species [5]. *L. catesbeianus* is native to eastern North America and has spread rapidly into more than 40 countries since the 19th century [6,7]. *L. catesbeianus* causes various types of damage to the indigenous environment. They are the vector to *Batrachochytrium dendrobatidis*, a fungal pathogen that can spread to native anurans, such as *Glandirana rugosa*, *Pelophylax nigromaculatus*, and *Rana dybowskii* [8,9,10]. Tadpoles in high density can alter nutrient cycling and ecosystems with high biomass intake, alter the algal communities, and depress the fish population [11,12,13]. *L. catesbeianus* outcompetes several native species for habitat and food resources and preys on many species due to their voracious appetite, reducing biodiversity, and declining population of native endangered species [14,15].

*L. catesbeianus* has caused severe environmental problems in South Korea. It was first introduced in South Korea in 1959 as a food source but failed to breed [16,17]. Reintroduction was made in 1971 to increase agricultural household income and captive breeding succeeded in the environment of South Korea in 1973, resulting in its nationwide distribution of farms [18]. Yet, the demand never reached an adequate level, and eventually, its population escaped or was released to nearby ponds, streams, and lakes [19,20,21]. In 1975, the survival of *L. catesbeianus* in the natural environment of South Korea was confirmed. Later, its negative effect on the environment became well known, and was eventually listed as invasive species by the Korean Ministry of Environment in 1998 [22,23].

Several management strategies were implemented to eradicate *L. catesbeianus* in South Korea. In 1997, the national government led control programs, including public eradication campaigns, advocacy as food sources, and monetary compensation to encourage *L. catesbeianus* capture [21]. Direct control was conducted to reduce the bullfrog population by removing eggs, trapping, and catching by hand. However, it is challenging to eradicate and manage *L. catesbeianus* once it is settled in the environment. The species is highly density-dependent, and insufficient control caused populational rebounds in past management projects [24]. Management without considering population size and dynamics is the leading cause of failed control, along with a lack of budget, time, and human resources [25]. Control plans should be based on a comprehensive understanding of population dynamics with biological and ecological knowledge to efficiently manage invasive species [26,27,28,29].

In this context, a dynamic population model may help us understand how and why the population changes over time [30,31]. The modeling approach is essential, especially in the case of species with stage structure and density dependency, such as *L. catesbeianus*, which can form complex dynamics [32]. In a dynamic population model, interactions between the species and its environment are simplified and represented with mathematical equations, sometimes in the form of diagrams. Therefore, one can evaluate and compare the efficiency of specific invasive species control methods by applying information about the control schemes to a dynamic population model [33,34,35]. A decision maker can draw a cost-efficient or optimal purpose-fit strategy to cull the population by analyzing population change in response to various control methods and intensities. Such a study may also provide scientific foundations and managerial insights to guide public opinions, which can lead to funding and the success of control management [36].

This study aims to suggest a practical control strategy for *L. catesbeianus* by estimating the population size and effectiveness of various methods and intensity control schemes. A dynamic population model of *L. catesbeianus* was built based on the ecological characteristics of each life cycle stage. STELLA architect software was used to build the model. Control methods currently implemented at the study site were parameterized and applied to the population model, and the results were analyzed and compared to evaluate their efficiencies.

## 2. Materials and Methods

### 2.1. Study Site

**Figure 1:**
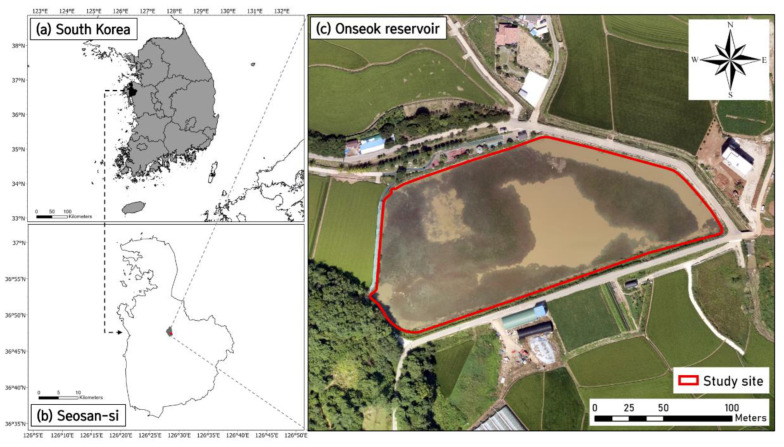
Map showing the location and red outline of the study site (Onseok reservoir). (**a**) South Korea, (**b**) Seosan-si, Chungcheong-namdo, and (**c**) Onseok reservoir.

The study site is the Onseok reservoir (36°47’41.5” N, 126°28’26.3” E), located in Seosan-si, Chungcheong-namdo, South Korea (Figure 1). The Onseok reservoir is used for irrigation, maintaining a constant water level throughout the years. With a constant water level, slow water flow, and plenty of hydrophytes for spawning, the reservoir is a suitable habitat for *L. catesbeianus*. According to the local residents, *L. catesbeianus* was first sighted in the Onseok reservoir in the early 2000′s. Since then, as the population has increased, intensive control methods utilizing double fyke nets and direct capture were implemented to reduce the number of individuals in 2021.

### 2.2. Collecting Environmental Data

**Figure 2:**
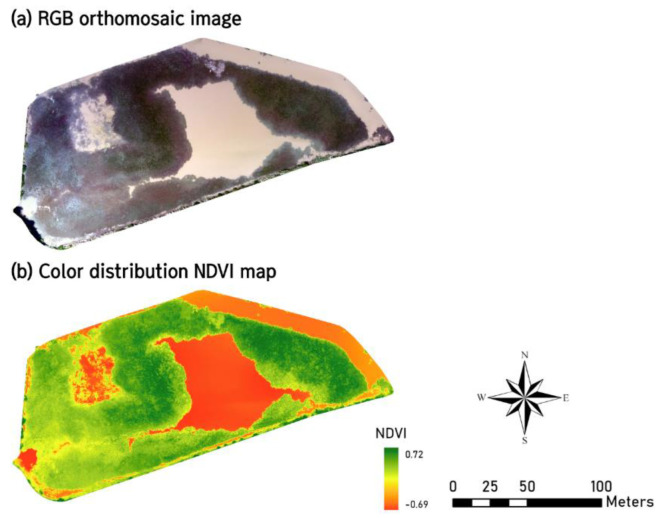
(**a**) Unmanned aerial vehicles (UAV)-derived RGB orthomosaic image of the study site. (**b**) Normalized difference vegetation index (NDVI) map.

We collected environmental data, such as the distribution area of hydrophytes and water surface area in the Onseok reservoir, to derive the carrying capacity of each *L. catesbeianus* life stage to build a site-specific model to guide further information in the ongoing management. A survey with unmanned aerial vehicles (UAV) was conducted on September 3, 2021, to calculate potential spawning and habitat area. DJI Phantom 4 RTK (DJI Technology Co., Ltd., Shenzhen, China), equipped with a multispectral sensor, was used to acquire RGB images and calculate the normalized difference vegetation index (NDVI) (Figure 2). Healthy vegetation reflects more near-infrared (NIR) and less red-light wavelength region [37]. NDVI is a spectral index quantifying vegetation by calculating a ratio difference between the reflectance measured in the NIR and red-light bands based on the reflectance properties of vegetation [38]. The NDVI values range from −1 to +1, with values closer to +1 indicating dense vegetation [39]. The spectral bands of the multispectral sensor used for NDVI calculation were 650 ± 16 nm for the red-light band and 840 ± 26 nm for the NIR band.

Female *L. catesbeianus* lay and anchor their eggs on hydrophytes. Therefore, the spawning area is associated with microhabitats with hydrophytes [40]. Hence, the distribution of hydrophytes was considered the spawning area. It was calculated by extracting a value of more than 0.3 based on the properties of moderate to dense vegetation with a positive NDVI value [41]. The water surface area was derived from the RGB image and used as the habitat area of the tadpole, juvenile, and adult stages.

### 2.3. Dynamic Population Model for L. catesbeianus

In our model, the initial population started with 10 adults, ready to reproduce. Individuals developed through egg, tadpole, juvenile, and adult stages. Population changes in the egg stage can be described as follows:

(1) $$P_{egg}\left(t\right)=P_{adult}\left(t-1\right)\times F,$$

where *P_egg_* (*t*) was the population of the egg stage, *P_adult_* (*t* − 1) was the population of the adult stage, and F was the fertility of *L. catesbeianus*.

The biological development time for tadpoles and juveniles in the environment in South Korea is 3 years [42]. A total of 7 years is required for a generation to develop into an adult fully. In the model, 3 years of delay were applied to describe the population change of tadpole and juvenile stage: the total number of tadpoles was equal to the number of existing tadpoles plus newly hatched tadpoles in the current year, minus the number of tadpoles that became juvenile in the current year. The equation for the tadpole population was as follows:

(2) $$P_{tadpole}\left(t\right)=P_{egg}\left(t-1\right)\times S_{egg}+\overset{2}{\underset{i=1}{\sum }}P_{tadpole}\left(t-i\right)-P_{tadpole}\left(t-3\right)-R_{fyke}\left(t\right),$$

where *P_tadpole_* (*t*) was the population of the tadpole stage, *P_egg_* (*t* − 1) was the egg stage population entering the tadpole stage, *S_egg_* was the survival rate of egg, *P_tadpole_* (*t* − *i*) was the population available at the tadpole stage, and *P_tadpole_* (*t* − 3) was the tadpole population moving to the juvenile stage.

We adopted the survival rate (*S*) based on the logistic growth equation of Verhulst [43]. S could be represented as follows:

(3) $$S=r\times \left(1-\frac{C\times P\left(t\right)}{K}\right),$$

where *r* was the intrinsic survival rate, *C* was the logistical regulation term constant, and *K* was the carrying capacity. The variables used in (3) were unique to each development stage, except for *K* for the juvenile and adult stages, which shared the same environmental niche.

Tadpoles can be controlled by sets of double fyke nets, known as one of the most effective control methods [44]. The number of tadpoles captured by the fyke nets in the current year *R_fyke_* (*t*) was described by the following equation:

(4) $$R_{fyke}=P_{tadpole}\left(t\right)\times \left(1-{\left(1-E\right)}^{N}\right),\;$$

where *P_tadpole_* (*t*) was the population size of the tadpole at time *t*, E meant the effectiveness of a single set, and N was the number of sets.

The equation describing the population of the juvenile stage was similar to (2). However, the control rate applied to juvenile and adult stages was given by the percentage of its population size since no literature information about control efficiency was available. The population of juvenile *P_juv_* (*t*) was described by the following equation:

(5) $$P_{juv}\left(t\right)=P_{tadpole}\left(t-3\right)\times S_{tadpole}+\left(1-R_{rate}\right)\times \sum_{i=1}^{2}P_{juv}\left(t-i\right)-P_{juv}\left(t-3\right),$$

where *R_rate_* was the control rate applied to both juvenile and adult stages. *P_tadpole_* (*t* − 3) was the tadpole population that entered the juvenile stage, *S_tadpole_* was the survival rate of tadpoles, *P_juv_* (*t* − *i*) was the population at the juvenile stage, and *P_juv_* (*t* − 3) was the juvenile population moving to the adult stage.

The population of adult stage *P_adult_* (*t*) could be described as follows:

(6) $$P_{adult}\left(t\right)=P_{juv}\left(t-3\right)\times S_{juv}+P_{adult}\left(t-1\right)\times S_{adult}\times (1-R_{rate}),$$

where *P_juv_* (*t* − 3) was the juvenile stage population entering the adult stage, *S_juv_* was the survival rate of juveniles, *P_adult_* (*t* − 1) was the population present at the adult stage, and *S_adult_* was the survival rate of adults.

### 2.4. Model Implementation

We utilized the STELLA architect software (isee systems, Inc.) to implement the population system dynamics model based on the above equations. STELLA architect software is a tool for building models of dynamic systems. The main components of the STELLA model are (1) stocks, where flowing variables are accumulated, (2) flow, which is the exchange among variables, (3) converter, which is an auxiliary variable controlling the variable and flow, and (4) connector, which is the connections between each modeling component. Users combine these components to build a model describing a dynamic system that forms a causal loop or a feedback structure of the phenomenon. A positive feedback loop is formed when the system output grows the system, and a negative feedback loop hinders the growth of the system [45]. By combining these two components, the model can express dynamic patterns, such as growth, reduction, convergence, and fluctuation of various systems [46].

**Figure 3:**
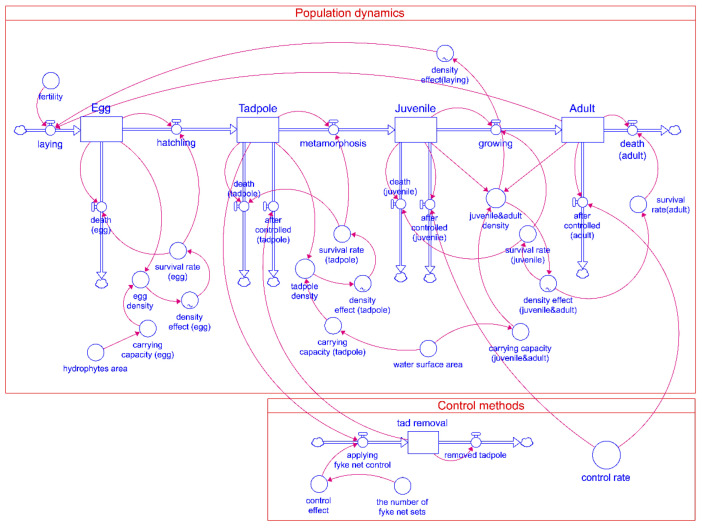
STELLA model for population dynamics of *L. catesbeianus* as affected by control methods.

We built the STELLA model to simulate the population dynamics of *L. catesbeianus* and to evaluate the effectiveness of the control (Figure 3). The first stage of the model simulates the population dynamics of *L. catesbeianus* and the environmental carrying capacity that limits growth. Stock represents the population size at each life stage. There are two flows. One is development flow, where the population transitions into the next life stage. The other is natural death flow, where the population fails to develop further. Generations pass through the egg, tadpole, juvenile, and adult stages, with respective parameters and time steps (Table 1). The main parameters are fertility, survival rate, carrying capacity, and control effect [14,27,28,47,48]. The survival rate was estimated based on a literature review and depended on population density. The population density was the ratio of the current population and the carrying capacity of the environment. We determined the carrying capacity based on a literature review, estimated by the surface area achieved through the UAV survey.

The control methods of *L. catesbeianus* were implemented in the control sector of the STELLA model. There were three control scenarios. In Scenario 1, tadpoles were controlled only by using sets of double fyke nets. In Scenario 2, juveniles and adults were controlled according to the control rate. In Scenario 3, Scenarios 1 and 2 were combined. The number of double fyke net sets ranged from 1 to 5. The control rate of juvenile and adult stages used in our model ranged from 0 to 0.8, with intervals of 0.2. The control effect was measured by calculating the mean of the remaining adult population from year 20 to 50, as the population stabilized after 17 years. Each scenario was simulated with 30 replicates.

## 3. Results

Population Dynamics of the Model for L. catesbeianus

**Figure 4:**
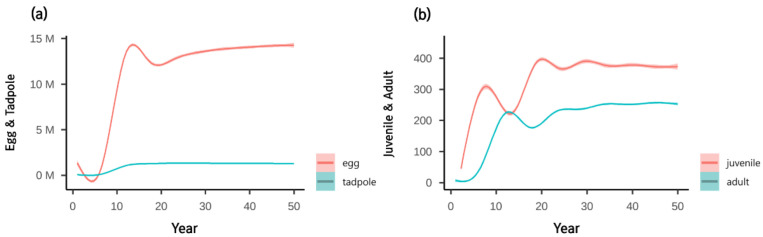
Long-term population dynamics of *L. catesbeianus* for (**a**) egg and tadpole stages and (**b**) juvenile and adult stages. Results show the mean and standard error (SE) with 30 replicates.

Population changes were simulated without control at each life stage (Figure 4). The population increases at the seven-year time step, as the initial population begins to reproduce from the first year. The population grew and stabilized after approximately 20 years. The egg and tadpole stages stabilized after reaching the peak, while the juvenile and adult stages continued to fluctuate in comparison. The adult population reached a maximum of 242 individuals (Table 2).

**Figure 5:**
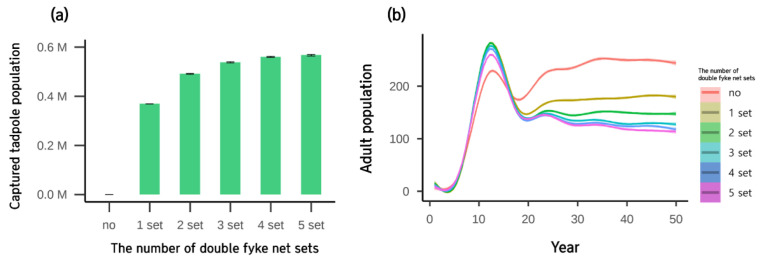
(**a**) The effect of the number of double fyke net sets on captured tadpoles during the simulation period and (**b**) adult population. Results show the mean and SE with 30 replicates.

Control efficiency was simulated under three scenarios. In Scenario 1 (tadpole control with sets of double fyke nets), more tadpoles were captured with high numbers of double fyke net sets, but capture efficiency decreased when more than four sets of double fyke nets were used (Figure 5a). The change in the adult population had a clear difference in control efficiency after 20 years (Figure 5b). Similar to tadpoles, the efficiency of double fyke net decreased as the mean population reduced only by three individuals when the number of sets increased from 4 to 5.

**Figure 6:**
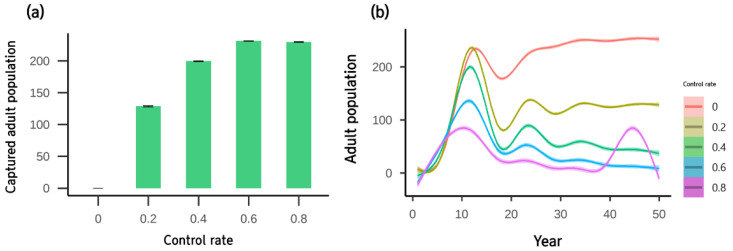
(**a**) The effects of the control rate on captured adults during the simulation period and (**b**) the adult population of each control rate over time. Results show the mean and SE with 30 replicates.

In Scenario 2 (direct removal of both juvenile and adult by the control rate), the number of captured adults did not increase when the control rate was higher than 0.6 (Figure 6a). The effect of the control rate on the adult population was evident for 10 years across all control rates used in the simulation (Figure 6b). The adult population at an 80% control rate had a sudden rebound pattern (Figure 6b), resulting in a higher mean population than the 60% control rate (Table 2).

**Figure 7:**
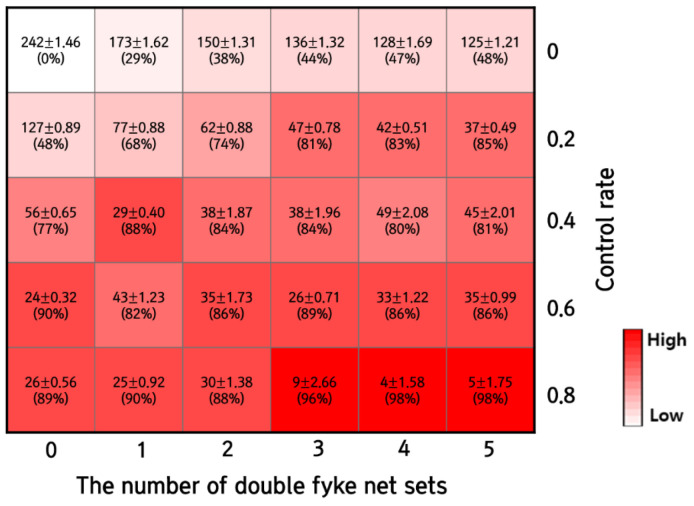
The number of the remaining adult population (mean ± SE) and reduction rate of *L. catesbeianus* after combining the two control methods.

The control effects of the adult population in Scenarios 1 and 2 differed significantly (Figure 7). While Scenario 1 was ineffective in adult population reduction (48% at best), the population reduced substantially (as much as 89%) in Scenario 2. Furthermore, when the control of tadpoles, juveniles, and adults was combined, the control effects increased dramatically in Scenario 3 (Figure 7). The reduction rate was 68%, even with the lowest control rate and the number of double fyke net sets, increasing to 98% with maximum control intensity levels. Only three sets of double fyke nets and a control rate of over 80% maintained an adult population under 10 individuals, which may be considered an acceptable level.

## 4. Discussion

We built a population dynamics model to evaluate the effectiveness of various control methods and their intensity. It was shown in our results that, without control, the adult bullfrog population reached 242 individuals and remained at such a level afterward. When only tadpoles were controlled (Scenario 1), the adult population decreased by half when five double fyke net sets were used. However, the efficiency of double fyke nets was limited in higher numbers, especially when more than three sets were used, which merely reduced the population by nine (Table 2). When juveniles and adults were removed (Scenario 2), the population decreased sharply with a higher control rate. In Scenario 3 (tadpole, juvenile, and adult control combined), the adult population decreased to a manageable level at a control rate of 80% and more than three double fyke net sets.

The modeled population without control increased dramatically after seven years (Figure 4a), which is the time required for *L. catesbeianus* to fully develop into the adult stage and start reproducing [49]. The population of the egg and tadpole stages stabilized after the peak before 20 years, as adults continuously reproduced with high fertility. This pattern agreed with the population dynamics that usually occur with introduced species, which initially grow rapidly and then decrease over time as carrying capacity, predators, and parasites take effect [50]. Juvenile and adult stages fluctuated in opposite directions as juveniles advanced to adult stages because they shared the same carrying capacity.

Among the control methods, the removal of eggs was not simulated in our model due to its lack of effectiveness [51]. Tadpole control by double fyke net was widely considered the most cost-effective method in small, shallow water bodies [48]. Direct control was applied to juveniles and adults, as no distinction was made when the management of the species was conducted in the field. A counterintuitive aspect of our result was that a higher control intensity did not always correspond with a population decrease. Such as a rebound pattern in the 80% control rate in Scenario 2 (Figure 6b) and in Scenario 3, where the remaining adult population tended to increase with more fyke net sets between 40% and 60% control rate (Figure 7). This pattern was derived from an overcompensatory survival rate corresponding to density dependence, especially from the tadpole stage, as the mortality in the early stage can increase the growth of adults [52,53]. The tadpole stage is highly density-dependent, and in high density the development rate decreases [54], but when partly removed, the survival rate can increase more than five times [55]. These population dynamics occasionally appear as a typical pattern in species with developmental stages and high fertility [56]. It has been reported that overcompensation is often found in *L. catesbeianus* control, as mortality increases by density dependence play a more significant role than the control agent [57]. Rosen and Schwalbe [58] showed how insufficient control methods could boost the growth and survival of the remaining population. In their study, adult removal was conducted three times. Nonetheless, after a few seasons, the population rebounded to 50–80% of the pre-removal population.

In Scenario 3, where the combined control of tadpoles, juveniles, and adults was adopted, the population decreased significantly, even with low-intensity control measures (Figure 7). The control rate of 80% combined with more than three sets of double fyke nets almost always resulted in a population decrease to an extremely low level (Figure 7). This result highlights the importance of population management across multiple life stages for species with complex life cycles. Our result agreed with the study of Guibert et al. [59]. These researchers reported that *L. catesbeianus* management in France was effective with direct control of the juvenile and adult stages and a combination of multiple tactics over continuous years. The study was one of the few successful eradication cases and mirrored the results of our research and the importance of integrated and continuous management [60].

Meanwhile, our model did not consider some of the ecological aspects that could influence the demographics of *L. catesbeianus* due to a lack of information. Such as the development rate of the tadpole stage to its density, which can strengthen the overcompensation of the adult population [28]. While empirical and local data can improve the performance of sophisticated population models, such data are rare, especially for species with complex life cycles [61]. The parameters used in the model are mainly based on a literature review of foreign study sites. As temperature and extreme weather conditions can significantly influence the development and growth period of *L. catesbeianus*, our parameters derived from different environments can be considered an important source of uncertainty in our results. This model can be improved by collecting more data on the life history traits of *L. catesbeianus* adapted to the local environment.

Past studies have been focused on finding pertinent development stages to target that can effectively reduce population size, as density dependence can hinder control effectiveness. Govindarajulu et al. [28] proposed to cull the one-year-old juvenile stage in the fall, which was the least sensitive stage to the density effect. Doubledee et al. [52] simulated the population change of *L. catesbeianus* by implementing intraspecific attacks among the development stages and showed that the density of the adult stage influenced the survival rate of the juvenile stage. Both studies implied the limitations of controlling only a single stage, leading to an increased survival rate in other development stages. We simulated population changes by applying single and simultaneous control methods. We successfully showed that simultaneous control of tadpole, juvenile, and adult stages effectively reduced the population to a manageable level.

The management strategies for invasive species should be based on the characteristics of the local environment and the status of their population dynamics [28,56]. Kim and Koo [25] pointed out that the failure of *L. catesbeianus* management in South Korea could be attributed to the absence of the coordination of species and site-specific control methods. This might occur because most controls were conducted non-systematically and briefly.

While limited in some aspects, our model was built based on the best available knowledge about the site-specific demographics and ecological characteristics of *L. catesbeianus*. The control scenarios applied to our model are readily applicable and can be useful in planning control strategies and evaluating the cost-effectiveness of invasive species management. We suggest that integrated, intensive, and continuous control is essential to effectively managing the population. Although the model was built based on the characteristic of the Onseok reservoir, we believe our suggestion on the control is universal as our result share similar results with research abroad. The population dynamics model could be a valuable tool for deriving efficient control strategies when data is less than perfect.

## 5. Conclusions

Understanding the population dynamics of invasive species plays a vital role in their management. The population dynamics model built in this study showed population variation over time, reflecting the biological and ecological characteristics of *L. catesbeianus*. Furthermore, the control scenarios were simulated to derive the efficiency of the control methods. Our results showed that implementing integrated controls throughout the development stages is the most effective.

## Figures and Tables

**Table 1:** Description of parameters used in the STELLA dynamic population model for each sector.

Model Sector	Parameter	Description	References
Population Dynamics	Population size	The population at each stage in the life cycle of *L. catesbeianus*	-
	Survival rate	The rate of survival is affected by the density effect in developing to the next stage	[28], [47]
	Fertility	The number of maximum eggs produced by female *L. catesbeianus*	[14]
	Carrying capacity	Maximum population size at each stage in the life cycle of the American bullfrog in the Onseok reservoir	[27], [48]
Control methods	Control rate	Rate of removed individuals to total population at each juvenile and adult stage	-
	The number of double fyke net sets	The number of the double fyke net set used to control	-
	Control effect	The control efficiency of the double fyke net set	[48]

**Table 2:** Variation of the adult population from year 20 to 50 by control method and intensity.

Control Method	Intensity	Population		
		Mean	SE	Range
No control	-	242	0.76	137
The number of double fyke net sets	1	173	0.50	91
	2	150	0.41	66
	3	136	0.41	74
	4	128	0.42	97
	5	125	0.46	126
Control rate	0.2	127	0.32	54
	0.4	56	0.46	69
	0.6	24	0.42	62
	0.8	26	1.04	154

## Data Availability

Not applicable.
